# Case Report: Tubal recanalization and short-term re-pregnancy after repeat ligation following two cesarean sections

**DOI:** 10.3389/fmed.2026.1958986

**Published:** 2026-09-29

**Authors:** Hui-Liu Fan, Bei Liu, Hong-Lan Wei

**Affiliations:** 1 Department of Gynecology, Guangzhou Women and Children’s Medical Center Liuzhou Hospital, Guangxi, China; 2 Guangxi Clinical Research Center for Obstetrics and Gynecology, Liuzhou, China

**Keywords:** cesarean section, contraceptive failure, re-pregnancy, tubal ligation, tubal recanalization

## Abstract

**Background:**

Tubal ligation is a widely used permanent female sterilization method. Laparoscopic tubal recanalization offers a pathway to restore natural fertility for women who desire pregnancy after sterilization. However, short-interval re-pregnancy following repeat tubal ligation after recanalization is clinically rare.

**Case presentation:**

We report a case of a 37-year-old woman, gravida 3, para 2, with a history of two previous cesarean sections, the second of which was accompanied by bilateral tubal ligation. Eight years after ligation, she underwent laparoscopic bilateral tubal recanalization due to remarriage and a renewed desire for childbearing. She conceived spontaneously 3 months postoperatively and delivered at 38 weeks via a third cesarean section, during which repeat bilateral tubal ligation was performed using the modified Pomeroy technique. Eleven months later, she conceived spontaneously again and underwent induced abortion due to no further fertility plans.

**Conclusion:**

Although pregnancy rates after tubal recanalization can reach 80%–90%, repeat ligation following recanalization does not guarantee absolute contraceptive efficacy. Spontaneous re-canalization with subsequent pregnancy remains possible. This case highlights the importance of standardized surgical technique, long-term follow-up, and postoperative contraceptive counseling, particularly in patients with a history of tubal surgery.

## 1 Introduction

Bilateral tubal ligation is one of the most commonly performed permanent female sterilization procedures worldwide (1). In China, concurrent tubal ligation during cesarean section is a frequent practice, as it accomplishes both delivery and sterilization in a single operation. However, spontaneous re-canalization resulting in pregnancy after tubal ligation, though rare, has been documented, with reported recanalization rates ranging from 0.2% to 2% (2).

For women who desire pregnancy after tubal ligation due to remarriage or other personal circumstances, tubal recanalization is an effective approach to restoring natural fertility (3). With advances in laparoscopic microsurgical techniques, laparoscopic tubal recanalization has progressively replaced conventional open surgery. Reported pregnancy rates after laparoscopic tubal recanalization range from 80% to 90% (4). Multiple factors affect the 1-year intrauterine pregnancy rate following hysteroscopic-laparoscopic tubal recanalization (5).

Theoretically, repeat tubal ligation after recanalization should provide reliable contraceptive efficacy; however, rare cases of subsequent pregnancy have been reported (6). What makes this case unique is the complete clinical course of “two cesarean sections with ligation → recanalization → pregnancy → repeat ligation during cesarean → short-term re-pregnancy” occurring within a single patient, providing a rare opportunity to examine the mechanisms and risk factors for contraceptive failure after repeat ligation. To our knowledge, no prior case report has documented this complete clinical trajectory.

## 2 Case description

### 2.1 Patient information

A 37-year-old woman, gravida 3, para 2, with a history of two previous cesarean sections, presented to our gynecology department with a desire for pregnancy. During her second cesarean section, she had undergone bilateral tubal ligation after completing her childbearing plan. Eight years after ligation, following remarriage, she sought to restore fertility (Table 1).

**Table 1:** Demographic and clinical data (de-identified).

Item	Data
Age at presentation	37 years
Gravida/Para	Gravida 3, Para 2
Medical history	Non-contributory (no hypertension, diabetes, or other chronic diseases)
Medication history	None
Family history	Non-contributory
Social history	Non-smoker, non-drinker

### 2.2 Preoperative evaluation

Preoperative assessment included ovarian reserve evaluation (serum anti-Müllerian hormone level **2.8 ng/mL**, antral follicle count **15**), pelvic ultrasound, and uterine scar assessment, all of which were within normal limits. No contraindications to surgery were identified.

### 2.3 Tubal recanalization and subsequent pregnancy

Laparoscopic bilateral tubal recanalization was performed under general anesthesia. The scarred tissue at the tubal isthmus ligation site was excised, and the tubal stumps were meticulously anastomosed using microsurgical technique with 5-0 monofilament sutures. Bilateral tubal patency was confirmed intraoperatively by chromopertubation. The procedure was uneventful, with an estimated blood loss of 30 mL and a total operative time of 65 min. The patient was discharged on postoperative day 2.

Three months after recanalization, the patient conceived spontaneously, confirmed by transvaginal ultrasound. The pregnancy was uneventfully monitored with serial ultrasound assessments of fetal growth and lower uterine segment scar thickness. She delivered at 38 weeks of gestation via a third cesarean section, during which repeat bilateral tubal ligation was performed. According to the operative record, the surgical team intended to perform a modified Pomeroy technique with excision of a 2–3 cm segment and burial of the proximal stump; however, the precise anatomical location of the ligation was not explicitly documented in the operative note. A healthy female newborn was delivered with Apgar scores of 9 and 10 at 1 and 5 min, respectively. The immediate postoperative course was unremarkable, and the patient was discharged on postoperative day 4.

### 2.4 Short-term re-pregnancy following repeat ligation

Eleven months after the third cesarean section, the patient presented with amenorrhea. Urine pregnancy test was positive, and transvaginal ultrasound confirmed an intrauterine gestational sac corresponding to 7 weeks of gestation. The patient had no further fertility plans and requested termination. Following counseling, she underwent suction evacuation, and the procedure was uneventful.

## 3 Discussion

### 3.1 Strengths and limitations of the case management

#### 3.1.1 Strengths

The patient received comprehensive preoperative evaluation, including ovarian reserve testing, before recanalization. The recanalization procedure was performed using standard microsurgical techniques with intraoperative patency confirmation. Postoperative follow-up was complete through the re-pregnancy event.

#### 3.1.2 Limitations

This case has several important limitations. First, hysterosalpingography was not performed after repeat ligation, which would have provided objective imaging evidence of tubal re-canalization and helped confirm the mechanism of contraceptive failure. We acknowledge that there are currently no established clinical guidelines recommending routine hysterosalpingography after tubal sterilization; nonetheless, in this specific case, we identified the lack of HSG confirmation as a limitation from an academic perspective, as it would have provided objective imaging evidence of tubal patency and helped clarify the mechanism of contraceptive failure. Second, histopathological examination of the excised tubal segments was not performed, preventing tissue-level confirmation of the specific re-canalization pathway (e.g., neocanalization vs. fistula formation). The operative records did not contain intraoperative photographs or detailed anatomical descriptions of the second ligation site; we recognize that this limits our ability to definitively determine the mechanism of contraceptive failure in this case. Third, the patient’s partner did not undergo repeat semen analysis at the time of re-pregnancy, which could have helped rule out other fertility factors. Finally, as a single case report, the findings are not generalizable to all patients undergoing similar procedures.

### 3.2 Mechanisms of pregnancy after tubal ligation

The fundamental pathological basis for pregnancy following tubal ligation is the inherent regenerative and remodeling capacity of fallopian tube tissue (7). Multiple retrospective analyses have systematically elucidated the mechanisms underlying failed tubal sterilization. A study of 39 women who became pregnant after tubal ligation reported the following causes: neocanalization in 19 cases (48.7%), fimbrial neogenesis in 4 (10.3%), tuboperitoneal fistula in 6 (15.4%), missed or incorrect ligation in 4 (10.3%), and tubal endometriosis in 2 (5.1%) (8). Retrospective studies have reported comparable etiological distributions for pregnancies following cesarean-concurrent tubal ligation, including tubal neocanalization, fimbrial neogenesis, tuboperitoneal fistula, and missed surgical ligation (6).

**These mechanisms can be categorized into three pathways (**9)**:**

1. **Recanalization Mechanisms—Neocanalization and Fimbrial Neogenesis.** Following ligation, epithelial cells at the tubal stump retain proliferative and migratory capacity, enabling the formation of microscopic channels that bypass the ligation scar. The regenerative potential of tubal epithelium constitutes the fundamental biological basis for contraceptive failure. Combined, neocanalization and fimbrial neogenesis account for 59%–67% of ligation failures.
2. **Fistula Mechanisms—Tuboperitoneal Fistula.** Abnormal fistulas may form between the ligated stump and the peritoneal cavity, allowing sperm to reach the distal tube or an ovum to enter the peritoneal cavity for fertilization. These account for 10%–15% of ligation failures.
3. **Operative Factors—Missed or Incorrect Ligation and Site Selection.** Some failures are attributable to technical issues such as loosened sutures, incomplete ligation, or misidentification of the round ligament. Notably, the ligation site critically influences failure risk: in one study, recanalization occurred at the ampulla in 8 of 12 cases (66.7%). The ampulla, with its wider lumen, abundant mucosal folds, and greater epithelial regenerative capacity, is predisposed to recanalization. In contrast, the isthmus has a narrower lumen, thicker muscularis, and lower regenerative potential. Therefore, isthmic ligation using the modified Pomeroy technique is currently recommended as the method with the lowest recanalization rate (10).

In the present case, the exact anatomical location of the second tubal ligation could not be definitively confirmed from the operative records, as the precise site was not explicitly documented. The operative record only indicates that a modified Pomeroy technique was intended. Given the patient's history of prior recanalization surgery—which inevitably results in tubal adhesions, anastomotic scarring, and altered anatomy—it is plausible that the normal isthmus was difficult to expose, potentially compelling the surgeon to ligate the ampulla instead, despite the intended isthmic approach. Alternatively, even if an isthmic ligation was successfully performed, the inherent regenerative capacity of tubal epithelium may have led to spontaneous re-canalization.

### 3.3 Clinical outcomes and influencing factors of tubal recanalization

Tubal recanalization is the primary approach for restoring fertility after ligation (3, 11). A study of 80 patients reported that recanalization was successfully achieved in 79 cases (12). Laparoscopic surgery has demonstrated superiority over open surgery: a comparative study of 66 patients reported significantly higher re-pregnancy rates in the laparoscopic group compared with the laparotomy group (*P* < 0.05) (13).

Factors influencing postoperative pregnancy rates include (14):

• **Anastomosis site:** Isthmic–isthmic anastomosis yields better outcomes than ampullary–ampullary or isthmic–ampullary anastomosis.

• **Duration of sterilization:** Longer intervals may lead to irreversible epithelial and muscular changes.

• **Patient age:** Advanced age is associated with diminished ovarian reserve and lower pregnancy rates.

• **Postoperative tubal length:** Longer preserved functional tubal length correlates with higher pregnancy rates.

In this case, conception within three months of recanalization confirmed the efficacy of laparoscopic recanalization and suggested favorable ovarian reserve and tubal condition.

### 3.4 Contraceptive failure after repeat ligation post-recanalization

The most distinctive aspect of this case is the short-interval re-pregnancy (11 months) after repeat ligation. Several factors may explain this phenomenon:

1. **Increased Technical Difficulty of Secondary Ligation.** After recanalization, fallopian tubes exhibit anastomotic scarring, luminal tortuosity, and peritubal adhesions, altering their original anatomy. During repeat ligation, the isthmus may be difficult to expose due to adhesions, forcing the surgeon to choose the ampulla as the ligation site. As noted above, ampullary ligation is associated with a higher recanalization rate.
2. **Variation in Contraceptive Efficacy by Ligation Technique.** Tubal sterilization methods include salpingectomy, tubal occlusion, and tubal ligation. Salpingectomy provides irreversible and definitive contraception. In contrast, ligation and occlusion preserve some degree of reversibility, and failure rates vary by technique. The modified Pomeroy technique has a lower failure rate than simple ligation or clip methods (10). Whether the most reliable technique was employed in this case is uncertain.
3. **Individual Variation in Tubal Regenerative Capacity.** Some women may have intrinsically higher tubal epithelial proliferative and regenerative potential, predisposing them to neocanalization even after properly performed ligation. This individual variability likely explains why the vast majority of women achieve permanent sterilization, while a minority experience recanalization within a short interval.

### 3.5 Comparison with similar and contrasting cases

Published clinical studies are summarized in Table 2.

**Table 2:** Comparison with similar and contrasting cases.

Study	Sample	Key Finding	Relevance to This Case
Analysis of 39 cases, 2010 [8]	39 cases	Neorecanalization most common cause (48.7%)	Supports mechanism in this case
Rekha V et al., 2026 [6]	60 cases	Sterilization failure rate 0.36%–0.42%; tubal recanalization accounts for 50%	Confirms recanalization as the leading cause of failure
Xiao M et al., 2018 [2]	Literature review	Recanalization rate 0.2%–2%	Provides baseline failure rate context
Feng QY, 2018 [12]	80 cases	Successful recanalization in 79 (98.75%); intrauterine pregnancy rate 80.00%	Confirms high success rate of primary recanalization

### 3.6 Take-away lessons from the case

1. **Standardize ligation technique:** During cesarean section, tubal ligation should be performed at the isthmus using the modified Pomeroy technique to minimize recanalization risk. For patients with previous tubal surgery, the isthmus should be carefully identified; if the isthmus cannot be adequately exposed due to adhesions, salpingectomy should be considered to ensure definitive contraception (15).
2. **Conduct thorough preoperative assessment:** Patients seeking recanalization should undergo comprehensive evaluation including ovarian reserve (with age as an independent predictor), semen analysis, and assessment of the original ligation site. If natural conception probability is low, assisted reproductive technology should be prioritized.
3. **Provide postoperative contraceptive counseling:** Pregnancy should be promptly excluded in patients presenting with amenorrhea, even after bilateral tubal ligation. For patients who have undergone repeat ligation following recanalization, enhanced postoperative surveillance and consideration of hysterosalpingography to confirm tubal patency are warranted.
4. **Adopt multidisciplinary management:** For patients with multiple cesarean sections combined with pregnancy after recanalization, a multidisciplinary team including reproductive surgery, obstetrics, and anesthesia should provide integrated care from preconception assessment through delivery.
5. **Consider salpingectomy for definitive contraception:** For women seeking permanent contraception who have risk factors for failure (e.g., history of tubal surgery, young age), bilateral salpingectomy may be a more reliable option than repeat ligation (15).

## 4 Patient perspective

The patient was informed about the report and its publication. She provided written informed consent and expressed the following perspective:

“I chose tubal recanalization because I wanted a chance to have a child in my new marriage. The surgery worked, and I am grateful for my baby. When I became pregnant again after the second ligation, I was truly surprised—I thought I had made a permanent decision. I hope sharing my story can help other women understand that even permanent procedures may not always be permanent, so they can make better-informed decisions with their doctors.”

## 5 Conclusion

This case demonstrates that while laparoscopic tubal recanalization is highly effective for restoring fertility, repeat tubal ligation following recanalization does not guarantee absolute contraceptive efficacy. Short-interval re-pregnancy can occur due to tubal neocanalization, anatomical changes from previous surgery, or individual regenerative capacity. Clinicians should employ the most reliable surgical technique (isthmic ligation with the modified Pomeroy method), provide comprehensive postoperative counseling, and consider salpingectomy for patients who desire definitive permanent contraception with elevated failure risk.

## Data Availability

The datasets presented in this study can be found in online repositories. The names of the repository/repositories and accession number(s) can be found in the article/Supplementary Material.

## References

[1] Peterson HB Xia Z Hughes JM. The risk of pregnancy after tubal sterilization: findings from the U.S. Collaborative review of sterilization. Am J Obstet Gynecol. (1996) 174(4):1161–70. 10.1016/s0002-9378(96)70655-0 8623843

[2] Xiao M Liu Y Li R Lu XH Huang ML Qin HY. Analysis of causes of pregnancy after tubal ligation. Chin J Fam Plann. (2018) 26(5):398–401.

[3] Gordts S Campo R Puttemans P. Laparoscopic tubal anastomosis: a review of the literature. Gynecol Surg. (2018) 15(1):1–8. 10.1186/s10397-017-1035-z 29375283 PMC5762801

[4] Yoon TK Sung HR Kang HG Cha SH Lee CN Cha KY. Laparoscopic tubal anastomosis: fertility outcome in 202 cases. Fertil Steril. (1999) 72(6):1121–6. 10.1016/S0015-0282(99)00425-2 10593394

[5] Jiang J. A study on influencing factors of pregnancy after hysteroscopic-laparoscopic tubal recanalization. Heilongjiang Med J. (2025) 38(2):426–9. 10.14035/j.cnki.hljyy.2025.02.062

[6] Rekha V Jayanthi K Saranya K Uthra KG. A three-year retrospective study of female sterilization failure at a tertiary care hospital. Int J Reprod Contracept Obstet Gynecol*.* (2026) 15(5):1618–24. 10.18203/2320-1770.ijrcog20261257

[7] Godin PA Syrios K Rege G Demir S Charitidou E Wery O. Laparoscopic reversal of tubal sterilization; a retrospective study over 135 cases. Front Surg*.* (2019) 5:79. 10.3389/fsurg.2018.00079 30687715 PMC6333701

[8] Ding ME. Analysis of 39 cases of tubal ligation failure. Med Inform (Mid-month*).* (2010) 5(12):3571. 10.3969/j.issn.1006-1959.2010.12.146

[9] Xue FX Lin A Wang Y. Expert consensus on the diagnosis and treatment of fertility-related chronic endometritis (2025 edition). Chin J Pract Gynecol Obstet. (2025) 41(7):715–22. 10.19538/j.fk2025070111

[10] American College of Obstetricians and Gynecologists. ACOG Practice bulletin No. 208: benefits and risks of sterilization. Obstet Gynecol. (2019) 133(3):e194–207. 10.1097/AOG.0000000000003111 30640233

[11] Hanafi M. Factors affecting the pregnancy rate after tubal microsurgery. Fertil Steril. (2003) 80(3):700–4. 10.1016/s0015-0282(03)00782-5 12909510

[12] Feng QY. Observation on the efficacy of recanalization for pregnancy after tubal ligation. Chin J Mod Drug Appl*.* (2018) (13):56–7. 10.14164/j.cnki.cn11-5581/r.2018.13.029

[13] Kim JH Lee YS Kim MR. Comparison of laparoscopic and laparotomic tubal anastomosis. J Korean Med Sci. (2004) 19(3):436–40. 10.3346/jkms.2004.19.3.436

[14] Liu FR Chen MH Zhao SH Bai WP. Application of random forest algorithm in pregnancy prediction after fallopian tube recanalization. Chin Gen Pract. (2025) 28(11):1361–6. 10.12114/j.issn.1007-9572.2023.0697

[15] Committee on Practice Bulletins—Gynecology. ACOG Practice bulletin No. 206: use of hysteroscopy for the diagnosis and treatment of intrauterine pathology. Obstet Gynecol. (2019) 133(2):e80–92. 10.1097/AOG.0000000000003062

